# Expression of Acetylcholine Receptors by Experimental Rat Renal Allografts

**DOI:** 10.1155/2014/289656

**Published:** 2014-07-09

**Authors:** Marion Meixner, Srebrena Atanasova, Winfried Padberg, Veronika Grau

**Affiliations:** Laboratory of Experimental Surgery, Department of General and Thoracic Surgery, Justus-Liebig-University Giessen, Feulgenstraße 10-12, 35392 Giessen, Germany

## Abstract

Chronic allograft injury (CAI) is a major cause for renal allograft dysfunction and characterized by vasculopathies, tubular atrophy, and fibrosis. We demonstrated that numerous leukocytes interact with vascular endothelial cells of allografts and produce acetylcholine, which contributes to vascular remodeling. The cholinergic system might be a promising target for the development of novel therapies. However, neither the cellular mechanisms nor the acetylcholine receptors involved in CAI are known. Kidney transplantation was performed in the Lewis to Lewis and in the Fischer-334 to Lewis rat strain combination, which is an established experimental model for CAI. Expression of nicotinic and muscarinic acetylcholine receptors mRNA was quantified in renal tissue by real-time RT-PCR on days 9 and 42 after surgery. We detected CHRNA2–7, CHRNA10, CHRNB2, CHRNB4, and CHRM1–3 mRNA in normal kidneys and in renal transplants. In contrast, CHRNA9, CHRM4, and CHRM5 mRNA remained below the threshold of detection. In renal allografts, CHRNA3 and CHRNB4 mRNA expression were dramatically reduced compared to isografts. In conclusion, we demonstrated that most acetylcholine receptor subtypes are expressed by normal and transplanted kidneys. Allograft rejection downmodulates CHRNA3 and CHRNB4 mRNA. The role of different acetylcholine receptor subtypes in the development of CAI remains to be established.

## 1. Introduction

Chronic allograft injury (CAI) is the most important cause of renal transplant failure in the long run, and up to now no effective therapies exist. CAI is associated with graft arterial intimal hyperplasia, glomerulopathy, interstitial fibrosis, and tubular atrophy [1]. The pathogenesis of CAI is poorly understood, but acute rejection episodes are a major risk factor and seem to trigger graft remodeling.

To gain insights into the pathogenesis of CAI, relevant experimental models are of utmost importance. Rat renal transplantation in the Fischer-344 (F344) to Lewis rat strain combination closely reflects human CAI [2–6]. Of note, a severe acute rejection episode peaks around day 9 posttransplantation and spontaneously resolves [7–10]. Six months after transplantation, however, renal function is impaired and all histopathological hallmarks of CAI are detected in allografts [8, 9]. This model opens up the possibility to investigate the contribution of acute rejection to CAI, which is difficult in patients. Using this experimental model, we observed numerous interactions of mononuclear intravascular leukocytes with endothelial cells of capillaries, veins, and arteries during acute rejection, which seem to contribute to the pathogenesis of allograft vasculopathies [7, 9].

These mononuclear allograft leukocytes express increased levels of the high-affinity choline transporter 1 (CHT1), mediating the uptake of choline into the cell, as well as increased levels of the choline acetyltransferase (ChAT), responsible for the synthesis of acetylcholine (ACh) [8]. Accordingly, authentic ACh was detected in these mononuclear leukocytes isolated from blood vessels of the grafts [8]. ACh receptor (CHR) activation plays a pivotal role in vascular remodeling [11–14] and in hyperproliferative disorders, which involve proliferation of fibroblasts and angiogenesis [15–21]. Hence, we postulated that leukocytic ACh contributes to the pathogenesis of CAI. As a proof of concept, we treated allograft recipients with the dual-specific choline esterase inhibitor rivastigmine, which should increase levels of endogenous ACh. Indeed, intimal hyperplasia was exacerbated in allograft recipients treated with rivastigmine [8]. Specific inhibition of cholinergic signaling between inflammatory blood leukocytes and graft blood vessels seems to be a promising approach for the development of novel therapies preventing CAI. In this context, detailed knowledge on the expression of CHR subtypes by healthy kidneys and renal grafts is needed.

Traditionally, CHR have been found in neurons and muscle cells, but over the last two decades it became evident that various nonneuronal cells express the CHR, among them are leukocytes and endothelial cells [22, 23]. Two classes of CHR have been described, nicotinic (CHRN) and muscarinic CHR (CHRM), named according to their prototypical agonists nicotine or muscarine. CHRN are ligand-gated ion channels, consisting of five subunits forming a cation selective ion channel, whereas CHRM are G protein coupled metabotropic receptors [24, 25]. In mammals, 16 nicotinic subunits (CHRNA1–7, CHRNA9-10, CHRNB1–4, CHRND, CHRNE, and CHRNG), which form either heteromers or homomers, as well as five CHRM (CHRM1–5) have been identified [26–29].

Our knowledge on CHR expression by rodent kidneys is scarce. In normal rat kidney tissue, CHRNA2, CHRNA3, CHRNA5, CHRNA7, CHRNA9, CHRNA10, CHRNB2, and CHRNB4 mRNA are detected [30]. Rat tubular epithelial cells express CHRNA2–7, CHRNA9, CHRNA10, and CHRNB2–4 mRNA. Among them, CHRNA2, CHRNA3, and CHRNA7 mRNA are most abundant [31]. In response to ischemia-reperfusion injury, which cannot be avoided during transplantation, tubular expression of CHRNA7 is quickly downregulated [30]. In mice renal interlobar arteries express the mRNA of CHRM1–5, with highest expression levels of CHRM3 [32]. However, data on CHR expression by renal transplant tissue are missing. Only for leukocytes isolated from the blood vessels of rat renal allografts undergoing fatal acute rejection, mRNA expression of CHRNA5, CHRNA9, CHRNA10, and CHRNB2 was described by our laboratory; expression of CHRM was not investigated [33].

In this study, we analyze the mRNA expression of a comprehensive set of CHR by normal Lewis kidneys, renal Lewis to Lewis isografts, and F344 to Lewis allografts on days 9 and 42 posttransplantation. Only receptors restricted to the neuromuscular end-plate were omitted from this study. We demonstrate that most CHRN and CHRM are expressed by all kidneys investigated and that the expression levels of some CHRN change in response to transplantation and/or rejection.

## 2. Material and Methods

### 2.1. Animal Experiments

Lewis and F344 male rats were purchased from Harlan Winkelmann (Borchen, Germany) and Janvier Labs (Le Genest Saint Isle Saint Berthevin, France). Animals were kept under conventional conditions until transplantation was performed at a weight of 270–300 g. Animal care and animal experiments were performed in accordance with current German animal protection laws as well as the NIH “principles of laboratory animal care.” Normal control kidneys were harvested from healthy untreated Lewis rats. Isogenic transplantation was performed in Lewis rats, whereas F334 rats served as donors and Lewis rats as recipients of allografts. Before transplantation, rats were anaesthetized with 60 mg/kg sodium pentobarbital (Narcoren, Merial, Hallbergmoos, Germany) intraperitoneally and donors were intravenously injected with 1000 U/kg heparin (Ratiopharm, Ulm, Germany) before removing the kidney. Kidney transplantation was performed as described before with minor modifications [34]. Shortly, kidneys were transplanted orthotopically to nephrectomized recipients and the ureter was anastomosed end-to-end. Warm ischemic times remained below 30 min. After surgery, recipients were treated with 150 mg ampicillin (Ratiopharm) intraperitoneally; no immunosuppression was applied. Nine and 42 days after transplantation, rats were anesthetized with sodium pentobarbital. Kidneys were removed immediately and cut into small pieces, which were snap-frozen and stored in liquid nitrogen until use.

To control the technical success of renal transplantation as well as allograft rejection, one slice of each transplant was embedded in paraffin. Sections of 5 *μ*m were stained with hemalum and eosin, azocarmine/aniline blue (Azan), or acidic orcein. Sections were evaluated with an Olympus BX51 (Hamburg, Germany) microscope.

### 2.2. Real-Time RT-PCR

Total RNA was extracted from 300 to 400 mg renal tissue (*n* = 4 for normal kidneys, isografts and allografts) at day 9 and day 42 after transplantation, using the RNeasy Miniprep Kit (Qiagen, Hilden, Germany) following the manufacturer's instructions. Reverse transcription (RT) was performed using 1 *μ*g RNA, MLV-RT, and random hexamer Primers (Promega, Mannheim, Germany). Alternatively, Superscripts II and III reverse transcriptases (Life Technologies, Darmstadt, Germany) were used. Thereafter, cDNA was analyzed in duplicate by quantitative real-time PCR with Platinum SYBR Green qPCR Super-Mix-UDG (Invitrogen, Karlsruhe, Germany) in an ABI 7700 Sequence Detection System (Applied Biosystems, Foster City, Canada). Negative controls were included in each experiment, where the template cDNA was replaced by water. Tongue and skin samples from healthy Lewis rats were used as positive controls. The program used for PCR included initial denaturation for 5 min at 95°C, followed by 45 cycles of 20 sec at 95°C, 20 s at 60°C, and 10 sec at 72°C, and a final extension step for 7 min at 72°C. Primers (MWG Biotech, Ebersberg, Germany) were designed to amplify intron-spanning sequences (Table 1) and were used at a concentration of 0.6 *μ*M. Melting curves of the PCR products were assessed; PCR products were analyzed by agarose gel electrophoresis and further verified by sequencing (Seqlab, Göttingen, Germany). Gene expression of CHRN and CHRM was normalized to the house-keeping gene porphobilinogen deaminase (PBGD) and calculated as arbitrary units by the delta delta CT method.

### 2.3. Statistical Analyses

Data were analyzed first by the nonparametric Kruskal-Wallis test and thereafter by the Mann-Whitney rank sum test using the SPSS software (Munich, Germany). Differences with *P* values below 0.05 were considered as significant. Two hypotheses were tested. (a) Transplantation changes CHR mRNA expression. To test this hypothesis, renal isografts were compared to normal healthy kidneys. (b) Rejection changes CHR mRNA expression. To test this hypothesis, renal allografts were compared to isografts an days 9 and 42 posttransplantation.

## 3. Results 

**Figure 1:**
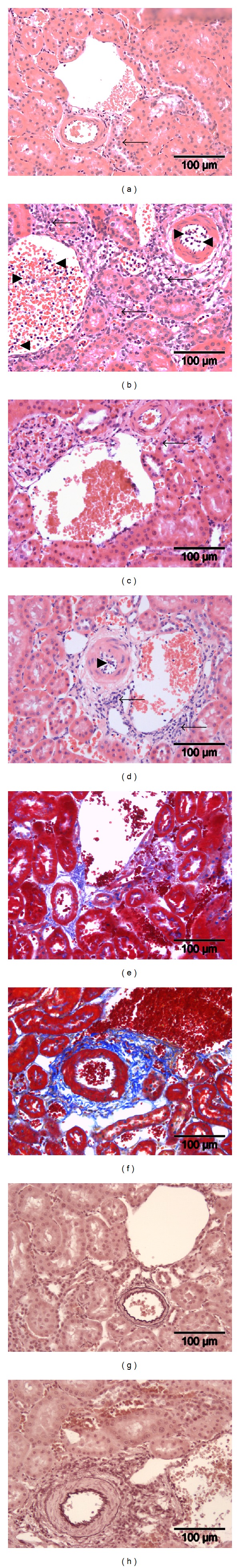
*Histopathology of renal isografts and allografts. *Hemalum and eosin-stained paraffin sections of renal isografts (a), (c) and allografts (b), (d) on days 9 (a), (b) and 42 (c), (d) posttransplantation are depicted, as well as sections of day 42 isografts (e), (g) and allografts (f), (h) stained with azocarmine/aniline blue (e), (f) and acidic orcein (g), (h). Arrows are pointing to perivascular infiltrates, arrowheads to intravascular leukocytes.

Histopathological changes caused by renal transplantation and acute allograft rejection were evaluated on paraffin sections of renal isografts and allografts on days 9 and 42 posttransplantation (Figures 1(a)–1(d)) and resemble previous data on the same experimental model [9]. The histomorphology of renal isografts was almost normal and only small mononuclear infiltrates were visible (Figures 1(a), 1(c), 1(e), and 1(g)). In contrast, day 9 allografts were strongly infiltrated by mononuclear leukocytes, which formed dense cuffs surrounding blood vessels and a diffuse infiltrate in the renal interstitium (Figure 1(b)). In the lumina of blood vessels numerous leukocytes were detected (Figure 1(b)). The renal parenchyma was largely unimpaired, and shedding of the tubular brush border was only observed in a minority of renal tubules. On day 42 after allogeneic transplantation, both the infiltrate and the number of intravascular leukocytes were markedly reduced, but discrete fibrotic changes were seen in perivascular regions (Figures 1(d) and 1(f)). Intimal hyperplasia typical for CAI, however, was not yet detected 42 days after surgery (Figure 1(h)). As described before, histopathological hallmarks of CAI such as vascular remodeling, interstitial fibrosis, and tubular atrophy develop in allografts within the following months [9].

**Figure 2:**
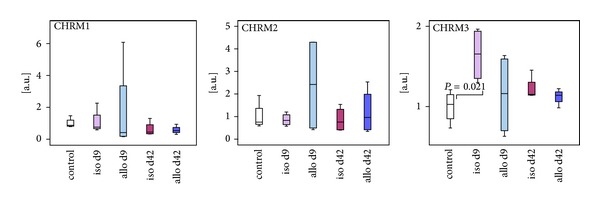
*Evaluation of mRNA expression of muscarinic acetylcholine receptors in control kidneys, isografts (iso), and allografts (allo) 9 and 42 days (d) after transplantation.* Real-time RT-PCR reveals expression of CHRM1–3 in all kidneys. Only CHRNA3 mRNA was transiently increased in renal isografts. Box plots indicate median and percentiles 0, 25, 75, and 100; *n* = 4 each.

We analyzed mRNA expression of CHRN and CHRM in normal kidneys, isografts, and allografts by real-time RT-PCR. In negative controls, no cDNA was amplified and in positive controls specific products of the expected size and sequence were obtained. In normal kidneys as well as transplanted kidneys, we found a prominent mRNA expression of CHRM1–3 (Figure 2), whereas the mRNA expression of CHRM4-5 remained below the threshold of detection. A slight and transient increase in CHRM3 mRNA was seen in day 9 isografts.

**Figure 3:**
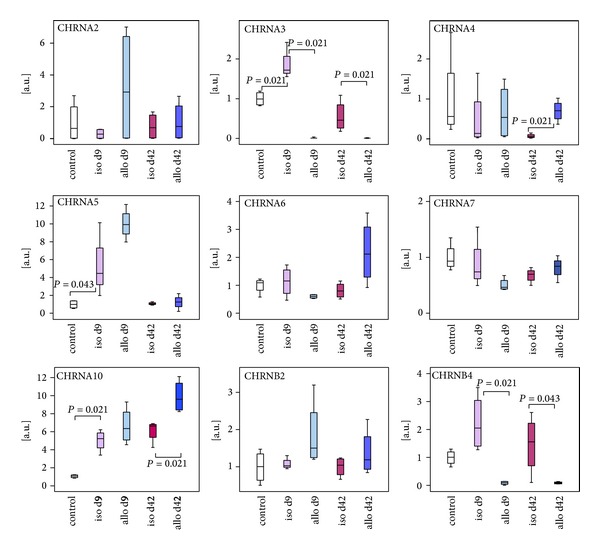
*Analysis of mRNA expression of nicotinic acetylcholine receptors by healthy control kidneys, isografts (iso), and allografts (allo) 9 and 42 days (d) after transplantation.* Real-time RT-PCR experiments reveal mRNA expression of CHRNA2–7, CHRNA10, CHRNB2, and CHRNB4 in all kidneys as well as differential expression of CHRNA3, CHRNA5, CHRNA10, and CHRNB4. Box plots indicate median and percentiles 0, 25, 75, and 100; *n* = 4 per group.

The mRNA of CHRNA2–7, CHRNA10, CHRNB2, and CHRNB4 was expressed in untreated kidneys, isografts, and allografts (Figure 3). The expression of CHRNA9 remained below the threshold of detection. CHRNA3 and CHRNB4 mRNA was strongly reduced in allogeneic kidney transplants both at day 9 and at day 42 when compared to isogenic transplants. We detected a transient transplantation-associated increase in the expression of CHRNA5 and CHRNA10 mRNA 9 days after transplantation, which went back to the initial levels 42 days after surgery (Figure 3). Furthermore, the expression of CHRNA10 and CHRNA4 was significantly increased in allografts at day 42 compared to respective isografts. The expression of all other analyzed CHRN and CHRM was similar in transplanted and untreated kidneys, both 9 and 42 days after transplantation.

## 4. Discussion

In this study, we demonstrate that the mRNA of most CHRM and CHRN is detected in normal rat kidneys, renal isografts, and allografts. The expression of some of these receptors is modulated in response to transplantation, which involves mechanical damage, ischemia/reperfusion injury, disruption of lymph vessels, and denervation. In addition, some changes only occur in allografts and are probably associated with allograft rejection. Acute allograft rejection is associated with accumulation of leukocytes in graft blood vessels, leukocytic infiltration of graft tissue, and organ damage on days 9 and 42 posttransplantation. In addition, the process of tissue remodeling, which eventually leads to CAI, is probably already taking place on day 42. All these transplantation associated changes might influence CHR expression.

CHRM1–3 are readily detected in all kidneys investigated. The transient increase in CHRNA3 mRNA in isografts but not in allografts probably reflects regeneration of perioperative damage, which might be less efficient in renal allografts. CHRM are expected to be expressed by smooth muscle cells of blood vessels and are involved in cholinergic vasorelaxation [35–40]. Gericke et al. [32] demonstrated that CHRM1–5 are expressed by rat renal interlobar arteries but only CHRM3 mediates the cholinergic vasodilation in rat kidneys [32, 41]. In our analysis, however, CHRM4 and CHRM5 mRNA remained below the threshold of detection, which might be explained by the fact that we worked on total renal tissue, whereas Gericke et al. [32] isolated renal interlobar arteries, where the expression of CHRM is probably higher compared to other parts of the kidney.

Among the CHRN investigated, only CHRNA9 was not detected in rat renal tissue. It is known that the detection of CHRNA9 is difficult and seems to require a specific reverse transcriptase [42]. Even though we performed the experiments with different reverse transcriptases (see Section 2.2), we were unable to detect renal CHRNA9 mRNA, whereas it was readily detected in rat tongue and skin, which served as positive controls. Previously, we reported expression of CHRNA9 by leukocytes residing in the blood vessels of renal allografts [33]. In addition, Yeboah et al. [30] described CHRNA9 mRNA expression by normal healthy rat tissue. We cannot explain the discrepancy between our finding and previous reports.

The mRNA of CHRNA5 and CHRNA10 was slightly increased in isografts and allografts in response to transplantation. This increase might reflect regeneration, graft infiltration by leukocytes, or induction of gene expression by inflammatory mediators. Concerning CHRNA10, it is most likely that the observed surgery- and rejection-associated increase in expression is due to leukocytic graft infiltration. CHRNA10 is typically expressed by monocytes and macrophages [23, 33], which accumulate in renal isografts and even more in allografts [7–10]. To the best of our knowledge, CHRNA10 does not form functional homomers but rather heteromers with CHRNA9 [43–46]. CHRNA9 mRNA can be expressed by mononuclear leukocytes, which accumulate in renal graft blood vessels during acute rejection [33] but remained below the threshold of detection in this study where total renal tissue was investigated. We conclude that CHRNA10 might form some functional heteromers with CHRNA9 but additional, possibly metabotropic, functions of CHRNA10 cannot be excluded. A slight and transient increase in CHRNA3 mRNA expression was seen only in isografts, which probably reflects graft regeneration rather than leukocyte infiltration.

In contrast, CHRNA3 and CHRNB4 were expressed at markedly lower levels in renal allografts compared to isografts. CHRNA3 and CHRNB4 are coexpressed and form functional receptor heteromers, which may also contain CHRNA5 [47–51]. Besides renal tubular cells, CHRNA3–5 were found in the immune system [23, 30, 52] and in the vascular endothelium [53, 54]. The allograft specific reduced expression of CHRNA3 and CHRNB4 mRNA might reflect damage of CHRNA3- or CHRNB4-positive cell populations, such as endothelial or tubular epithelial cells, caused by acute rejection or a downregulation induced by rejection-associated mediators.

Of note, we did not observe changes in CHRNA7 expression, which might have been expected since this receptor is downregulated by tubular epithelial cells in response to ischemia/reperfusion injury [31]. However, in contrast to the study of Yeboah et al. [31], which investigated changes in CHRNA7 expression within the first 24 hours after ischemia/reperfusion, our analysis was performed 9 days after surgery and tubular CHRNA7 expression might have been restored in between.

Our study has numerous limitations. The functional implications of the observed differences in CHR expression between allografts and isografts are difficult to predict. CHR are probably involved in vascular remodeling and graft fibrosis [11–21]. Considering the eminent role of ACh in immunity, changes in CHR expression might modulate the production of immune mediators and leukocyte migration [55–59]. Furthermore, we only investigated CHR expression on the mRNA level in total tissue samples and hence we ignored protein expression, cellular localization, and functionality of graft CHR. Analyses of CHR protein are still limited due to the lack of specific antibodies to CHRM and CHRN [60–63]. We made an attempt to detect CHRN7, CHRN9, and CHRN10 with labeled *α*-bungarotoxin and failed, probably due to low receptor expression by nonneuronal cells. In future studies, single-cell RT-PCR as well as in situ hybridization should be performed to identify cell specific changes in CHR expression. This knowledge is needed to design future therapies aiming at the prevention of CAI.

In conclusion, we demonstrated that most CHRN and CHRM are expressed by normal and transplanted kidneys and that a lack in receptors composed of CHRNA3 and CHRNB4 as well as an increase in receptors containing CHRNA10 might be involved in the pathogenesis of CAI. More studies are needed to define CHR expression in renal transplants on the cellular level.

## Figures and Tables

**Table 1:** Primer sequences used for real-time PCR.

Gene	Accession number	Direction	5′-3′ sequence	Product (bp)
CHRM1	NM_080773	Forward	tgtggccagcaacgcctctg	106
		Reverse	ccttcggggagtgcgcttgg	

CHRM2	NM_031016	Forward	gcccaacccaccacgagcc	102
		Reverse	agttgtggccgctgcggaa	

CHRM3	NM_012527	Forward	gtccctcggaggcagggct	116
		Reverse	acccccgagagggtcgctg	

CHRM4	NM_031547	Forward	cgactcgcggaacctctggc	117
		Reverse	ggcgtgaagttggccatgctg	

CHRM5	NM_017362	Forward	ccacagcaaagtcgatgaggca	102
		Reverse	tttggcctcccctcttcttggc	

CHRNA2	NM_133420	Forward	cacggccagtgcccaacact	119
		Reverse	ctgctttagccagacattggtggt	

CHRNA3	NM_052805	Forward	tgggtgttgtgctgctcccg	124
		Reverse	actggaacaggcggtgctca	

CHRNA4	NM_024354	Forward	agggaccggcctcttgcctg	113
		Reverse	tgcctactggccgagaccac	

CHRNA5	NM_017078	Forward	accagctgatgacgacgaacg	117
		Reverse	acagggagtccgaaggaacacg	

CHRNA6	NM_057184	Forward	ctttgagttggccatcacgc	116
		Reverse	ccgttggatcccaacacaatc	

CHRNA7	NM_000746.4	Forward	agatggccagatttggaaacc	142
		Reverse	gcaggaactcttgaatatgcct	

CHRNA9	NM_022930	Forward	atctggtgtggaggccggaca	119
		Reverse	ggccggtgagtcccaggtgat	

CHRNA10	NM_022639	Forward	accagtggcagatacagaccagac	124
		Reverse	tgtccactcttgccggatccac	

CHRNB2	NM_019297	Forward	gtccggctcccttccaaacaca	114
		Reverse	gctgccatcataggagaccacagc	

CHRNB4	NM_052806	Forward	ccgcctggagctatcactgtcc	110
		Reverse	tccaggccaggcggtagtca	

PBGD	NM_013168	Forward	ggcgcagctacagagaaagt	115
		Reverse	agccaggataatggcactga	
